# Factors influencing the recruitment of lactating women in a clinical trial involving direct oral anticoagulants: a qualitative study

**DOI:** 10.1007/s11096-018-0734-5

**Published:** 2018-10-10

**Authors:** Yating Zhao, Amally Ding, Roopen Arya, Jignesh P. Patel

**Affiliations:** 1 Institute of Pharmaceutical Science, King’s College London, 5th Floor Franklin-Wilkins Building, 150 Stamford Street, London, SE1 9NH UK; 2 King’s Thrombosis Centre, Department of Haematological Medicine, King’s College Hospital NHS Foundation Trust, London, UK

**Keywords:** Anticoagulation, Breastfeeding, Clinical trials, Direct oral anticoagulants, Thrombosis, Venous thromboembolism

## Abstract

*Background* Robust human data on medication use during lactation is scarce. With increasing medication use in postpartum women, it is important to conduct clinical lactation studies measuring the excretion of drugs in human milk and generate evidence. We plan to conduct a clinical lactation study, involving the direct oral anticoagulants (DOACs). *Objective* This study aimed to identify factors influencing lactating women’s clinical trial participation and to improve the design of a proposed DOACs clinical lactation study. *Setting* Lactating women in London, UK. *Methods* Three focus groups were conducted in lactating women with differing experiences of being prescribed anticoagulants during puerperium. *Main outcome measures* Thematic framework approach was used to analyse and identify key themes, using NVivo version 11. *Results* Eight breastfeeding mothers participated. Women’s decision-making on clinical trial participation was largely influenced by the lactation stage and previous breastfeeding experience. The concern of harm to their infant caused by the test medication or interruption of lactation were the predominant barriers to potential participation. Around 6 months following the birth of their infant and second-time mothers appeared to be more amenable to clinical trial participation. The provision of home visits for the execution of the study was highly recommended. *Conclusion* Our findings suggest that lactating women would participate in a clinical trial during the breastfeeding period, if the timing is right and if the woman is an experienced mother. Home visits will be provided in our proposed DOACs clinical lactation study.

## Impacts on practice

- Recruiting mothers approaching weaning stage and second-time mothers in clinical lactation studies is likely to lead to recruitment compared to mothers at early lactation stage and first-time mothers.
- The provision of home visits, where possible, may improve the recruitment of lactating women in clinical lactation studies.

## Introduction

Mothers are encouraged to exclusively breastfeed infants for the first 6 months of life, and continue up to 2 years with supplemental foods due to the benefits of breastfeeding to both infants and mothers [1]. It is estimated that the prevalence of receiving breastmilk for up to 6 months is 34% in the UK, 49% in the US and 71% in Norway [2]. During this period, more than 50% of postpartum women required at least one medicine [3]. Many health professionals may advise these women to discontinue breastfeeding due to concerns about potential risks to their infants [4]. Such advice is often not based on evidence, as human data on medication use during lactation is scarce [5–8]. The paucity of human data raises difficulties for physicians in making evidence-based recommendations on medications prescribed to women who want to breastfeed. Thus there is a need to conduct prospective clinical lactation studies, which measure actual drug concentrations in human milk, a view endorsed by regulatory authorities worldwide [9–11].

The risk of venous thromboembolism (VTE) during the puerperium increases dramatically [12–15]. In the UK, pulmonary embolism (PE) has remained a leading direct cause of maternal death for over 20 years [16, 17].VTE prophylaxis is recommended in large numbers of postpartum women and for most women after caesarean delivery by many national obstetric guidelines [18]. Currently, only heparins and warfarin have been demonstrated as safe anticoagulants during lactation [19]. Postpartum low molecular weight heparin (LMWH) has the disadvantage of having to be administrated as an injection, and in some women adherence to LMWH drops during the postpartum period compared to the antenatal period [20]. The use of warfarin is challenging as it exhibits considerable variability in dose response, thus requiring patients’ frequent attendance to clinics. The direct oral anticoagulants (DOACs), have many advantages over traditional anticoagulants, and have the potential to be prescribed for women during lactation [21, 22]. However, there is limited data regarding the extent of excretion of DOACs into human breast milk, and the clinical implications of DOACs for nursing infants, therefore they are contraindicated during lactation [23–25].

We plan to conduct a clinical lactation study to evaluate the excretion of rivaroxaban and dabigatran into human milk. Before we embark on this study, we wanted to understand women’s decision-making when invited to participate in a clinical trial whilst breastfeeding. Issues around clinical trial recruitment have been explored in other settings by conducting focus groups with potential participants, e.g. oncology [26], and it has been demonstrated as an effective way to help researchers design and conduct their proposed clinical trial [27]. To the best of our knowledge, no similar study has been done in lactating women before.

## Aim of the study

This study aims to identify factors influencing lactating women’s clinical trial participation and to improve the design of a proposed DOACs clinical lactation study.

## Ethics approval

Ethics approval was obtained from South West—Cornwall & Plymouth Research Ethics Committee (17/SW/0015), United Kingdom.

## Methods

### Study sample

We purposefully sampled mothers (Age ≥ 20 years and ≤ 45 years) who had breastfed within the past 5 years or were currently breastfeeding to participate in 3 focus groups. The three focus groups comprised of different populations: focus group 1 (FG1): mothers who had never been prescribed an anticoagulant for the prevention or treatment of VTE during the puerperium; focus group 2 (FG2): mothers who were at risk of VTE during pregnancy and/or the puerperium and injected LMWH during the puerperium; focus group 3 (FG3):mothers either with a clinical diagnosis of VTE and being prescribed LMWH during pregnancy and/or the puerperium or being prescribed long-term treatment with anticoagulation for the secondary prevention of VTE.

### Data collection and recruitment

FG1 mothers were recruited via www.mums.net. FG2 and FG3 mothers were identified through outpatient clinic letters from King’s College Hospital. Two different recruitment strategies were applied to maximise the participants for the different groups. The participant information leaflets (PIL) were mailed to eligible patients. Interested mothers actively contacted the lead researcher (YZ).

The focus groups were conducted at King’s College Hospital, London, UK between April and June 2017. A semi-structured topic guide (Table 1) formed the basis of the discussions. An experienced researcher (JPP), facilitated the discussions in the focus groups. The Lead Researcher (YZ) attended all focus groups, acted as a recorder and audio-recorded all sessions. Each focus group lasted approximately 1 h. Written informed consent was obtained from all participants. All data were securely stored to ensure confidentiality, privacy, and auditability. Recruited women were given a £30 retail voucher for their participation.

**Table 1:** Focus group topic guide

**Understanding of “clinical trial”**
1. What comes to mind when you hear the word: clinical trial?
**Breastfeeding women’s participation in a clinical trial**
2. Would you be willing to participate in a clinical trial whilst you are breastfeeding?
*Explore reasons*
3. What would make you feel more comfortable about participating in a clinical trial whilst breastfeeding?
4. What barriers do you think would keep you from participating in a clinical trial whilst breastfeeding?
**Perceptions of information flow regarding proposed DOACs clinical lactation study**
*(A PIL of the proposed DOACs clinical lactation study was given to participants prior to discussion; They were given 5* *min to read)*
5. What comes to your mind, when you read the information sheet for the proposed study?
*Generate discussion*
**Suggestion to the proposed DOACs clinical lactation study**
6. What would you say are things that would improve the proposed study?
7. Would you be willing/interested to participate in the proposed study if you were eligible to participate?
*Explore reasons*

### Data analysis

A thematic framework method was applied to analyse data [28]. All audio recordings were transcribed verbatim, and destroyed after being transcribed. Data were entered into NVivo version 11 (QSR International Pty Ltd, Melbourne, Australia), and systematically analysed in 5 steps. First, 2 members of the research team (YZ and AD) read and coded each transcript independently. After the initial open coding, the researchers compared codes and resolved disagreements by consensus. Second, the lead researcher refined and verbally defined the codes. Similar codes were clustered and given an initial category label. An analytical framework was created using an iterative process based on discussions among the entire group. Finally, the final analytical framework was applied to each transcript. Larger themes encompassing the categories were identified and described. The entire research team reviewed the results, and validated the interpretations in a final discussion.

## Results

A total of eight mothers were recruited across the 3 focus groups; 2 for FG1, 5 for FG2, and 1 for FG3. All mothers were between 26 and 45 years of age, currently breastfeeding. The characteristics of each participant are summarised in Table 2.

**Table 2:** Participant characteristics

ID	Maternal age (range in years)	Education level	Employment	Annual income from employment	Current infant age (months)	Current breastfeeding regime
A1	26–35	Post-graduate	Full-time	> £60,000	4	Breast only
A2	36–45	Post-graduate	Full-time	> £60,000	4	Breast only
B1	26–35	College	Full-time	> £60,000	3	Breast only
B2	36–45	Post-graduate	Full-time	£11,500–£31,999	7	Breast only
B3	26–35	Post-graduate	Full-time	£32,000–£59,999	7	Breast with occasional supplement^a^
B4	26–35	High school	Full-time	£32,000–£59,999	2	Breast with supplement at every feed
B5	36–45	College	Full-time	£32,000–£59,999	5	Breast only
C1	26–35	Post-graduate	Un-employed	< £11,500	13	Breast only (started to add solid food)

^a^Supplement included formula milk or any other solid food

### Perceptions of “clinical trial”

Mothers in all 3 groups had basic understanding about what a clinical trial was and entails. They defined a clinical trial as testing of new medication to cure disease and research based on professional evidence.

### Decision-making process

All mothers indicated that making the decision regarding clinical trial participation whilst breastfeeding was complicated. They would search for reassurance on the safety of the drug to their breastfed infants via other researchers, interaction with other mothers or online resources before making the decision. One mother used her experience of being prescribed a LMWH (enoxaparin) during lactation as an example:

I was constantly asking them (the doctors)…All the research I came across online, pointed towards it being alright and also, I knew other breastfeeding mums that were on precautionary doses of enoxaparin following caesareans so it’s quite standard that people take. So, I was reassured. (FG3, C1)

Women’s decision-making was also influenced by the stage of lactation. The majority of mothers (N = 7) appeared to be more comfortable with participation towards the ‘weaning’ stage of breastfeeding:

It’s not a crazy idea to ask women who are getting towards the end of their breastfeeding journey (to take part in clinical trials). Then you wouldn’t have to worry about having to breastfeed or trying to maintain a supply over the night feed. (FG1, A1)

Most women (N = 7) felt more relaxed to make decision with their second child:

I think I am more relaxed the second time around and I feel like as long as I’ve got milk brilliant and then let’s go and if it stops then go with the bottle and you know he might not take it to start with but we will manage. (FG2, B2)

In general, all mothers expressed that decision-making ultimately depended on weighing up the pros and cons from clinical trial participation.

### Motivations for clinical trial participation

Mothers across the 3 groups all indicated the possible benefits of positive health outcomes and for some, altruism through participation. The mother in FG3 discussed the potential health benefit to her in the future specific to participation in the proposed clinical trial:

I would absolutely jump at the chance (of participating in the proposed study) because I know for a second baby I would need to inject from day one, …so to take a pill instead would be so much easier. (FG3, C1)

Another significant motivation was the appreciation of healthcare providers who provided good care to mothers in the past. Mothers in FG2 discussed how the good care they received from the haematologists at King’s College hospital would motivate them to participate, whereas they might not be that motivated if asked by a research team who they were not familiar with:

I wanted to help because I had a really good experience with … and … and thought they really helped me out and I would like to do something. Whereas maybe, I don’t know it sounds a bit selfish really, someone I didn’t know, the health visitor I wouldn’t really be interested in a trial just because you know …that team (the research team of the proposed DOACs lactation study) was really good so I would like to do something if I can to help them out. (FG2, B4)

Mothers in FG1 also regarded financial incentives as important features through participation. As one mother indicated:

I’m not sure how many women would do it unless there was a financial motivation. (FG1, A2)

Incentives covering child care cost were also reported as being important by several mothers (N = 4) across the groups.

### Barriers for clinical trial participation

The most commonly reported barrier to participation were safety issues, particularly what the harms to infants might be. Many mothers (N = 7) indicated “child comes to the top” whilst breastfeeding, which brought extra cautions of safety to these mothers regarding participation:

I’d be slightly concerned going into a clinical trial in case there were adverse effects, especially if it’s, like, on a baby. (FG1, A1)

I think I definitely feel more risk adverse since having children and you have to look after yourself for their sake. (FG2, B2)

The baby would always come first. (FG3, C1)

Furthermore, participation was influenced by factors associated with lactation. All mothers reported that they would not interrupt breastfeeding their infants for participating in a clinical trial during the early lactation stage, unless they had a life-threatening condition which required the test drug. They were aware of the benefits of maintaining breastfeeding and believed that milk supply would be reduced by interrupting breastfeeding:

Those first few weeks where you’re trying to get to grips with the breastfeeding, it can be very difficult, so it’s having the support there for the mother. Because it’s tricky to be able to do the breastfeeding in the first place, and then to sort of put that in with the trial as well. (FG3, C1)

Despite the fact that a pump could be used to maintain breastfeeding during a clinical trial, a number of mothers mentioned that infants may refuse to bottle feeding and refuse to take frozen milk. Also, some mothers felt uncomfortable using a pump. A mother indicated that her breastfed infant would not drink the milk if it was frozen or if it had been stored in advance as “when I tasted my breast milk it was horrible”. (FG1, A2)

However, these factors appeared to reduce towards the ‘weaning’ stage due to less concerns on maintaining breastfeeding and reduced infant need for breastfeeding. In terms of the ‘weaning’ stage, mothers in FG1 mentioned a time-point of “6 months”, when they “switch to formula” and “start introducing solid food”:

I think a lot of it happens at around six months, because then that’s when you’re more likely to wean the baby on to food. And so you’re giving them different things anyway, so then that feels like a natural time to start introducing formula. But that’s also when they start to get more distracted. So he’d pull off when we were out and about and look around and it was just hard to try and make him feed whilst he was facing my breast, it was almost impossible. So giving him a cup of milk was a lot easier. (FG1, A2)

Concerns on childcare whilst participating in a clinical trial were also frequently mentioned (N = 7), especially for those who had no close families around and had more than 1 child:

I don’t have any family nearby, so it’s quite a big problem. (FG2, B5)

Practicalities from participation were also significant barriers, such as the pressure of time, accommodation in clinic, over-night stay and disincentives. Some mothers in FG1 and FG2 (N = 2) indicated that the pressure of time was a key barrier for their participation. A common negative aspect reported by all mothers was that the over-night stay brought inconveniences due to concerns on infants’ need for breastfeeding at night:

When she was in a particularly bad mood, I was just feeding her for comfort and she was falling asleep on my boob. Which I wouldn’t normally do, but she was just so miserable otherwise and I guess if you can’t give them that comfort during some difficult times that would be really hard. (FG1, A2)

Other barriers reported were the health status of participants, untrustworthy study researchers, and having a family to care. Some mothers (N = 5) expressed that healthy volunteers would be less interested in clinical trial participation because they could not receive health benefits from participation and would be more concerned about safety:

I wouldn’t want to take even a small chance that in the future they could potentially find a problem, and its different if I had a medical condition and this might be of benefit to you then I would probably be more interested than if I was just a healthy volunteer. I wouldn’t want to gamble even a small amount. (FG2, B4)

### Suggestions for the proposed clinical trial

One of the most prevalent concerns was the 24-h stay in the research site. Provision of home visits was suggested by nearly all the mothers (N = 7) across the groups, as it solved barriers associated with time, childcare and practicalities:

I think that would just take away travel time and there would be more resources for you and I think it would make it easy of women participating who can’t sort out the practicalities. (FG2, B2)

If they were required to stay in the research site over-night, the majority of mothers in FG1 and FG2 (N = 5) suggested having the infant accompany during the stay and the provisions of a silent, child-friendly environment and convenient facilities, whereas other mothers preferred staying without infants when towards the ‘weaning’ stage of breastfeeding. Some mothers (N = 4) suggested childcare support from researchers or incentives covering childcare.

In terms of milk sampling methods, some mothers (N = 2) suggested to have the right of either choosing hand expression or breast pumps:

Pumping is a very weird experience and a strange thing to do and something I don’t think probably want…the amount of milk you’ll want could probably be hand-expressed. (FG1, A1)

Furthermore, the majority of mothers (N = 7) expressed a preference for real-time results in terms of drug concentration in their milk, and follow-up procedure, before restarting breastfeeding to reassure themselves that there was no chance of infants being exposed to the test drug:

If you could run tests on the breast milk, and just say for example these are the results during the study, these are the results after, and the results before, so you know there’s absolutely nothing in the system, then you would continue breast feeding. (FG3, C1)

Some mothers (N = 4) suggested that second-time mothers and mothers who had used pumps should be actively recruited:

You’ll get a greater uptake from women who are already expressing or rather that will just be more likely to be more successful with women who are happily already pumping. (FG1, A1)

Most mothers (N = 5) seemed to be positive towards participation in the proposed study if they were motivated enough. Although several mothers (N = 3) were still concerned about their infants refusing to have pump milk or bottle feed, their attitudes would change if they were asked to participate towards the ‘weaning’ stage. One mother said:

For me personally that you came to see me at six months, I would take part in it. (FG3, C1)

## Discussion

In general, results in this study resonated to those from previous studies in other settings [26, 29–31]. Our findings, however, indicate that lactating women were more concerned about potential risks involved in clinical trials to their breastfed infants than to themselves, and in many ways, is an extension of what is seen during the antenatal period [30]. The risks involved were not only treatment side effects, but also risks to infants if they had to interrupt breastfeeding for clinical trial participation. Therefore, the decision-making process for clinical trial participation among lactating women was multi-layered, in comparison with that in other settings [26].

One significant barrier influencing participation was associated with the stage of lactation. The results suggest that many women are unlikely to interrupt breastfeeding at the early stage of lactation. Indeed, the early weeks of lactation was a critical period for the establishment of exclusive breastfeeding [32]. Interestingly, a new finding from the present study was that mothers were more likely to participate at around 6 months, when they started introducing supplemental food, and when infants had reduced physical and psychological need for breastfeeding.

Increased recruitment is likely to be seen in second-time mothers. This was understandable because first-time mothers had more pressures in terms of establishment and maintenance of breastfeeding and might feel unconfident about their abilities to nurture their infants, and additionally participate in a trial [33–35]. Conversely, experienced mothers felt more relaxed and flexible with breastfeeding, and additionally were less likely to maintain breastfeeding the second time round [36].

Our findings suggest that patients with severe medical conditions which might be managed by the test drug appeared to be more likely to take part in clinical trials during the lactation period, particularly at the early stage of lactation. That was because this population was not only motivated to take part through altruistic reasons but personal benefit. Another reason was the appreciation to the research team who provided good care to them in the past. Personal benefit of a positive health outcome as a motivation of clinical trial participation has also been seen in cancer settings [37].

Regarding the proposed trial, mothers suggested providing home visits instead of staying overnight at the research site. Bringing the conduct of clinical trial assessments to participants is a definite way to improve patient recruitment and retention [38]. A recent breastfeeding study successfully used home visits to improve the recruitment and retention of lactating women [39]. Once home visits were applied, various barriers of participation were less of a concern.

To the best of our knowledge, this qualitative study is the first to explore and describe lactating women’s opinions about clinical trial participation. We investigated women’s motivations and barriers toward clinical trial participation, as well as opinions on the proposed DOACs clinical lactation study by conducting 3 focus groups. The main limitation of our study is the small sample size, particularly FG3, where only 1 participant enrolled due to the limited number of lactating women who meet the inclusion criteria of this group. However, in-depth discussion was established between the coordinator and this participant in FG3. Additionally, thematic data saturation was reached, as 80% of codes were identified from FG1, with no new themes emerging by the completion of FG3 [40, 41]. Our work has important implications for clinical lactation research. Early data suggest that small amount of DOACs are excreted in human breast milk [23]. If this is confirmed by our proposed trial, future studies should be undertaken to definitively determine whether the amount of DOACs ingested by nursing infants is safe, which will require larger studies to be completed during breastfeeding.

## Conclusion

This study provides valuable information on the recruitment of lactating women in a clinical lactation study involving DOACs. The findings suggest that lactating women approximately 6 months into breastfeeding and second-time mothers are more likely to participate in a clinical trial, and the provision of home visits where possible, was seen as important.
